# BST2 regulates interferon gamma-dependent decrease in invasion of HTR-8/SVneo cells via STAT1 and AKT signaling pathways and expression of E-cadherin

**DOI:** 10.1080/19336918.2019.1710024

**Published:** 2020-01-20

**Authors:** Sonam Verma, Amandeep Kaur Kang, Rahul Pal, Satish Kumar Gupta

**Affiliations:** a Reproductive Cell Biology Laboratory, National Institute of Immunology, New Delhi, India; b Immunoendocrinology Laboratory, National Institute of Immunology, New Delhi, India

**Keywords:** IFN-γ, BST2, trophoblast invasion, E-cadherin, STAT1, AKT

## Abstract

The mechanism by which interferon-gamma (IFN-γ) downregulates trophoblast invasion needs further investigation. Treatment of HTR-8/SVneo cells with IFN-γ led to a decrease in their invasion concomitant with an increased expression of BST2. Silencing of BST2 by siRNA showed a significant increase in their invasion and spreading after treatment with IFN-γ as well as downregulated expression of E-cadherin. Further, STAT1 silencing inhibited the IFN-γ-dependent increase in the expression of BST2 and E-cadherin. Treatment of HTR-8/SVneo cells with IFN-γ led to the activation of AKT, and its inhibition with PI3K inhibitor abrogated IFN-γ-mediated decrease in invasion/spreading and downregulated BST2 and E-cadherin expression. Collectively, IFN-γ decreases the invasion of HTR-8/SVneo cells by STAT1 and AKT activation via increased expression of BST2 and E-cadherin.

## Introduction

Invasion of trophoblast cells is one of the crucial steps for successful implantation. Shallow invasion of the trophoblast cells is associated with pregnancy-related complications like preeclampsia (PE) and intrauterine growth restriction, and excessive invasion of the trophoblast cells leads to placenta accreta [1,2]. The levels of cytokines, growth factors, and chemokines present in the uterine environment secreted by the trophoblast cells and/or endometrium are important for trophoblast invasion [3,4]. The balance between the positive and negative regulators of invasion is vital for successful implantation. Interferon-gamma (IFN-γ) is one of the key cytokines, secreted by uterine natural killer cells and trophoblast cells during early and late pregnancy [5–7]. IFN-γ inhibits invasion of the trophoblast cells by altering the levels of different transcription factors and effector proteins [8,9]. The increased level of IFN-γ has been associated with many pregnancy-related complications like PE, miscarriages, and recurrent spontaneous abortions, and has also been implicated in failures of *in vitro* fertilization [10–14]. Thus, it may be pertinent to delineate the effector proteins and signaling pathways that are responsible for IFN-γ-mediated decreased invasion of trophoblast cells as observed during PE.

Bone marrow stromal antigen 2 (BST2), also known as CD317/tetherin/HM1.24 antigen, is a type II transmembrane glycoprotein known to be induced by IFNs [15,16]. BST2 is involved in pre-B cell growth, acts as an inhibitory factor of human immunodeficiency virus-1 replication, and also restricts the release of different enveloped viruses such as ebola virus, vesicular stomatitis virus,, and herpes simplex virus from the infected cells [17–20]. The cytoplasmic tail of BST2 can interact directly or indirectly with different effector proteins and regulate their functions [21,22]. Further, several studies have shown that overexpression of BST2 is also associated with tumor progression in different cancers like oral cavity, breast, and endometrial cancer [23–25]. However, there are reports which also show inhibitory effect of BST2 on the cell growth and motility of HT1080 (human fibrosarcoma epithelial cell line) and MDCK cells (Madin–Darby canine kidney cells [26]). Being a transmembrane protein, BST2 regulates different signaling pathways like NF-κB, PI3K/AKT, and ERK [27,28]. Moreover, it has been shown that the expression of BST2 is also regulated by the TLR4/AKT signaling pathway in macrophages [29]. Subsequently, studies have shown that expression of BST2 is dependent on unphosphorylated-signal transducer and activator of transcription 1 (U-STAT1) in BJ fibroblasts, hTERT-HME1 mammary epithelial cells, and non-tumorigenic human cell lines [30]. Further, the expression and promoter activity of BST2 are also controlled by signal transducer and activator of transcription 3 (STAT3) in tamoxifen-resistant breast cancer cells [31]. In our previous study, next-generation sequencing revealed an increased expression of BST2 in HTR-8/SVneo cells treated with IFN-γ for 24 h [9]. Since BST2 is known to be involved in invasion, migration, and growth of different cancer cells, it would be interesting to find out the role of BST2 in IFN-γ-dependent invasion of the trophoblast cells.

In addition to the JAK/STAT1 signaling pathway, IFN-γ also activates PI3K/AKT signaling pathway [32,33]. Activation of the AKT signaling pathway by IFN-γ helps in the maintenance of intestinal epithelial homeostasis by regulating beta-catenin (β-catenin) expression as observed in T84 cells [34]. Moreover, IFN-γ-induced GTPase contributes to the invasion of *Listeria monocytogenes* into the giant trophoblast cells by promoting the PI3K/AKT signaling pathway in mouse trophoblast stem cell line [35]. The importance of the AKT signaling pathway in regulating trophoblast invasion in the presence of IFN-γ has not been explored. However, there are studies which showed that AKT signaling pathway is activated by epidermal growth factor, hepatocyte growth factor, and human chorionic gonadotropin hormone and promotes invasion and migration of the trophoblast cells [36–39]. On the other hand, there are reports which also show that AKT inhibits migration and invasion of breast cancer cells by promoting proteasomal degradation of nuclear factor of activated T-cells (NFAT) transcription factors [40].

The invasion of trophoblast cells occurs with the contribution of different epithelial–mesenchymal transition (EMT) markers like cadherin and vimentin [41]. Studies have shown that the expression of E-cadherin is essential for embryonic development [42,43]. E-cadherin knockout mice are unable to form functional trophectoderm and thus could not survive during implantation [42]. Moreover, a decrease in the expression of E-cadherin has been reported in trophoblast cells during EMT when extravillous trophoblasts (EVTs) migrate or invade into the cell column [44].

In this study, we sought to elucidate the functional significance of BST2 in the regulation of trophoblast invasion in the presence of IFN-γ. Using matrigel matrix invasion assay, we studied the importance of BST2 and AKT signaling pathway in the IFN-γ-mediated decrease in invasion of HTR-8/SVneo cells as well as the importance of AKT signaling pathway in regulating BST2 levels. Further, considering the significance of STAT1 in IFN-γ-mediated decreased invasion, the levels of BST2 have also been investigated after silencing of STAT1. As E-cadherin plays an important role during invasion of trophoblast cells, its level in HTR-8/SVneo cells was also studied after silencing/inhibition of BST2 and STAT1 & AKT signaling pathways.

## Results

### BST2 plays an important role in IFN-γ-dependent decrease in invasion/spreading of HTR-8/SVneo cells

**Figure 1.**
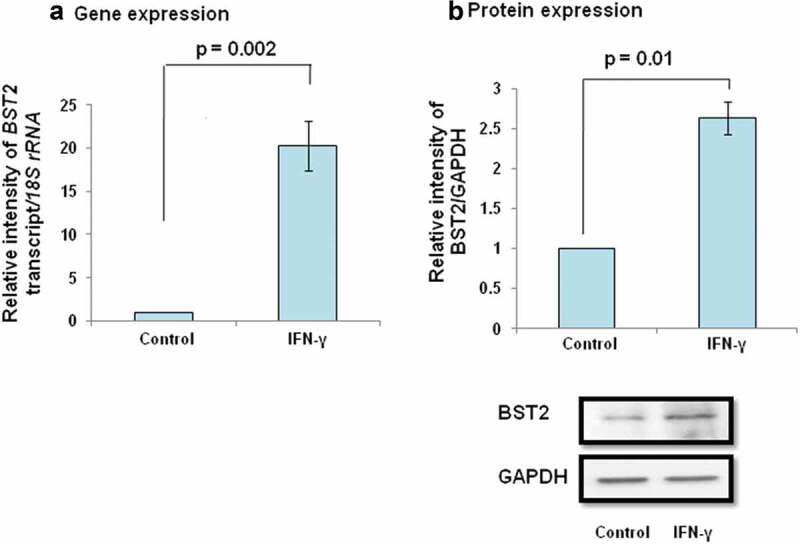
Expression of BST2 in HTR-8/SVneo cells subsequent to treatment with IFN-γ. HTR-8/SVneo cells (0.1 × 10^6^/well) seeded in 6-well culture plate were incubated overnight at 37°C in 5% CO_2_ and 70% relative humidity. The next day, cells were treated with IFN-γ (10 ng/mL) for 24 h as described in *Materials and Methods*. Subsequently, RNA followed by cDNA synthesis and cell lysates were prepared to study the expression of BST2 by qRT-PCR and Western blotting. Panel a shows the transcript level of BST2 in IFN-γ-treated HTR-8/SVneo cells with respect to its untreated control. Panel b shows the expression of BST2 at the protein level in IFN-γ-treated HTR-8/SVneo cells in comparison to untreated control. The representative blot from one of the three experiments is appended below the Panel b. For gene and protein expression, *18S rRNA* and GAPDH have been taken as normalizer. Values are expressed as mean ± SEM of three independent experiments, and p-value was calculated by using one-way ANOVA.

**Figure 2.**
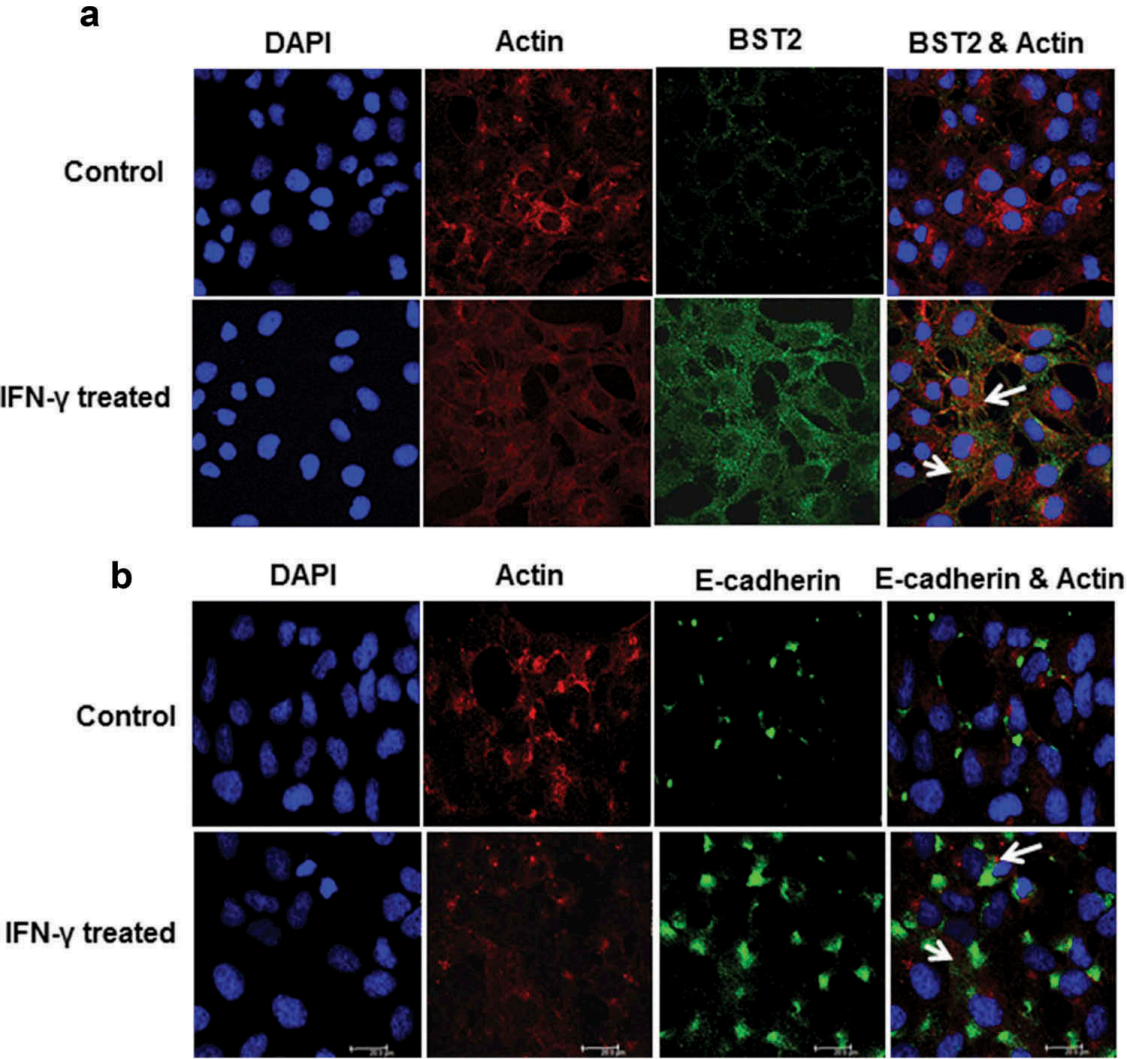
Immunofluorescent profile of BST2 and E-cadherin in HTR-8/SVneo cells treated with IFN-γ. HTR-8/SVneo cells (20,000/well) were seeded on the coverslips in 24-well cell culture plates and cultured overnight at 37°C in 5% CO_2_ and 70% relative humidity. The next day, cells were treated with and without IFN-γ for 24 h followed by immunolocalization of BST2, E-cadherin, and actin as described in *Materials and Methods*. Panel a shows the expression of actin (red) and BST2 (green) in control and IFN-γ-treated HTR-8/SVneo cells. Panel b shows the expression of actin (red) and E-cadherin (green) in control and IFN-γ-treated cells. The nuclei were stained using Hoechst nuclear staining dye. Scale bar shows 20 μm.

**Figure 3.**
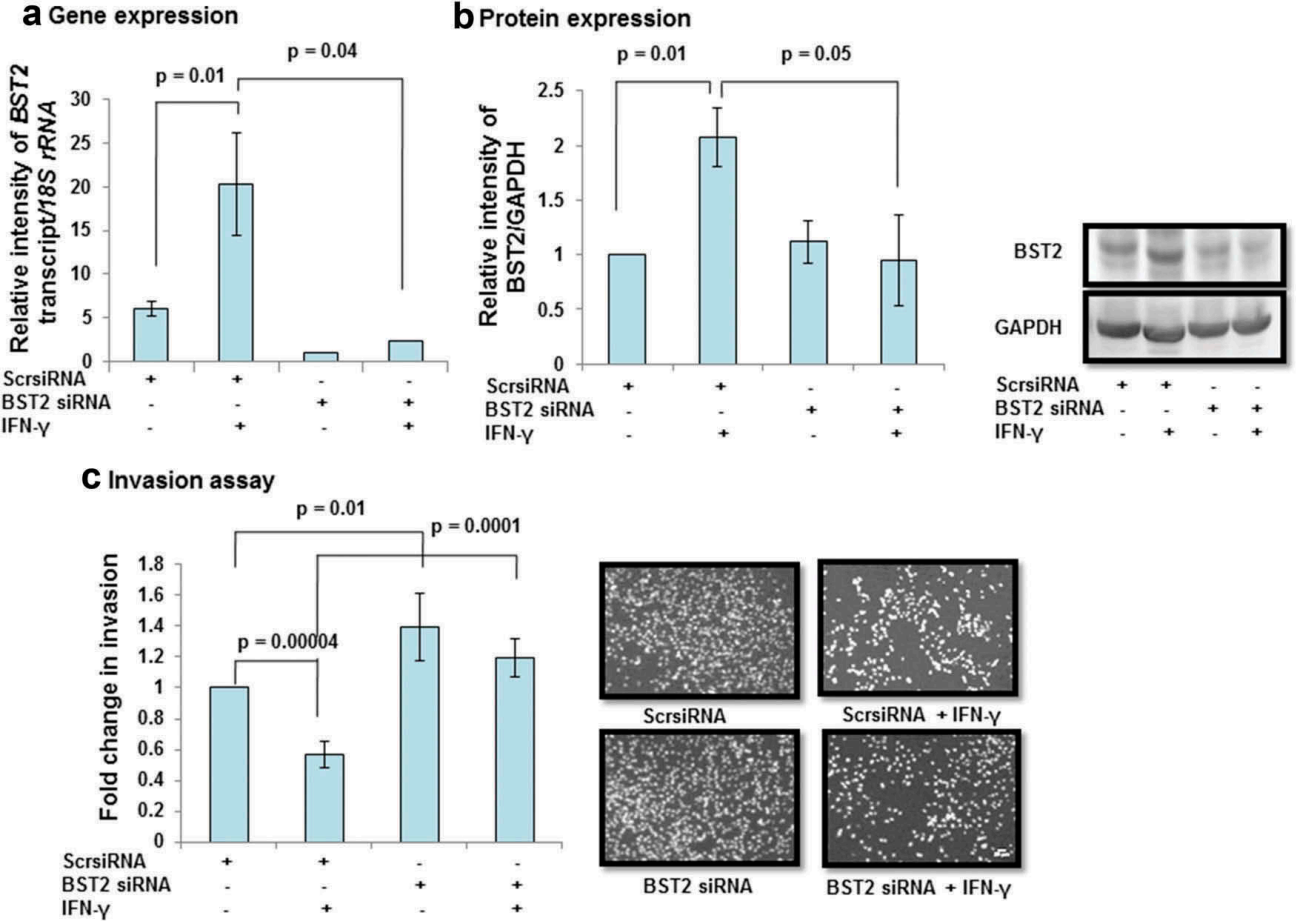
Effect of BST2 silencing on the invasion of HTR-8/SVneo cells treated with IFN-γ. HTR-8/SVneo cells (0.1 × 10^6^/well) were seeded in 6-well culture plate and cultured overnight at 37°C in 5% CO_2_ and 70% relative humidity. The next day, cells were transfected with scrambled siRNA or BST2 siRNA and subsequently used to study their invasion by matrigel matrix invasion assay as described in *Materials and Methods*. Silencing of BST2 was confirmed by qRT-PCR and Western blotting. Panels a and b show transcript and protein levels of BST2 in scrambled siRNA and BST2-silenced cells, respectively, on treatment with and without IFN-γ (10 ng/mL). The representative blots of BST2 and GAPDH from one of the three experiments are appended with Panel b. Each bar represents relative expression after normalization with *18S rRNA* or GAPDH and expressed as mean ± SEM of three independent experiments. Panel c shows the fold change in the invasion of cells transfected with scrambled or BST2 siRNA, respectively, followed by treatment with and without IFN-γ for 24 h. The representative images of the cells in the invasion assay are also appended with Panel c. The results are expressed as mean ± SEM of three independent experiments. Fold change in invasion is expressed with respect to scrambled siRNA-transfected cells without treatment with IFN-γ.

BST2, a type II transmembrane protein, is known to be involved in the invasion of cancer cells by regulating different signaling pathways and effector proteins [23–25]. In our previous report, next-generation sequencing data revealed the increased expression of BST2 in IFN-γ-treated HTR-8/SVneo cells at 24 h as compared to untreated control cells (GEO accession number - GSE125778; 9). Therefore, it would be interesting to delineate the functional significance of BST2 during the invasion of HTR-8/SVneo cells in the presence of IFN-γ. To study its importance, cells were treated with IFN-γ for 24 h, and expression of BST2 at transcript and protein levels was confirmed by qRT-PCR and Western blotting. The transcript level of *BST2* was significantly (p = 0.002) upregulated in IFN-γ-treated cells vs untreated control cells (Figure 1, Panel a). Similarly, the protein level of BST2 was significantly (*p* = 0.01) upregulated in IFN-γ-treated cells vs untreated control cells (Figure 1, Panel b). Further, IFN-γ-mediated expression of BST2 and its interaction with cytoskeleton protein, actin were studied by indirect immunofluorescence as described in *Materials and Methods*. Increased expression of BST2 and its interaction with actin were observed after treatment of HTR-8/SVneo cells with IFN-γ (Figure 2, Panel a). To establish the importance of BST2 during trophoblast invasion, BST2 was knocked down by using siRNA, and confirmation of its silencing was done by qRT-PCR and Western blotting. We have found a significant decrease in the expression of *BST2* transcript (~88%; *p* = 0.04) and protein (~50%; *p* = 0.05) levels after its silencing in BST2 siRNA-transfected cells vs scrambled siRNA-transfected cells in the presence of IFN-γ (Figure 3, Panels a and b). After confirmation of silencing, BST2-silenced cells and scrambled siRNA-transfected cells were used for matrigel matrix invasion assay. Inhibition of BST2 expression by siRNA led to a significant increase in the number of invading cells *vs* scrambled siRNA-transfected cells (~1.4 fold; p = 0.01) in the absence of IFN-γ treatment. Further, the invasive ability of BST2-silenced cells following treatment with IFN-γ was also significantly (~2.1 fold; *p* = 0.0001) increased with respect to scrambled siRNA-transfected cells in the presence of IFN-γ (Figure 3, Panel c).

**Figure 4.**
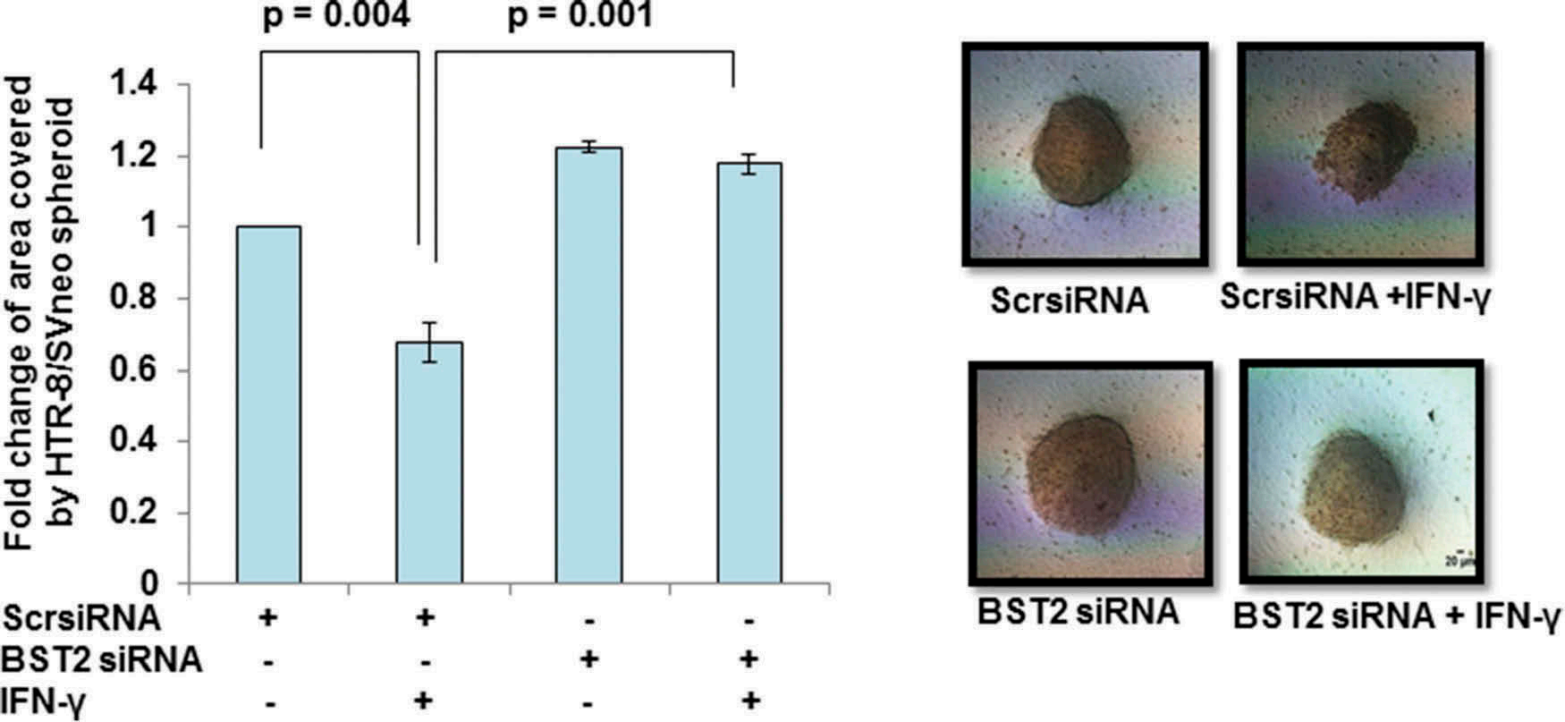
Spreading of BST2-silenced HTR-8/SVneo spheroids on the monolayer of Ishikawa cells. HTR-8/SVneo cells (0.1 × 10^6^/well) were seeded in 6-well cell culture plate overnight at 37°C in 5% CO_2_ and 70% relative humidity. The next day, cells were transfected with BST2 siRNA and scrambled siRNA and kept for 48 h followed by the formation of spheroids using 2500 cells/spheroid as described in *Materials and Methods*. The histogram shows the fold change in the area covered by individual scrambled siRNA-transfected HTR-8/SVneo spheroids and BST2-silenced HTR-8/SVneo spheroids, respectively, in the presence/absence of IFN-γ after 24 h. Each bar represents a relative area of spreading after normalization with untreated scrambled siRNA-transfected HTR-8/SVneo spheroids. The representative images of the spheroids are appended. Scale bar represents 20 μm. Values are expressed as mean ± SEM of three independent experiments.

Further, to understand the EVT–endometrial epithelium interface during early pregnancy, co-culture of HTR-8/SVneo spheroids after BST2 silencing onto the monolayer of Ishikawa cells was performed in the presence or absence of IFN-γ as described in *Materials and Methods*. There was a significant increase in the area covered by individual BST2 silenced HTR-8/SVneo spheroids on the monolayer of Ishikawa cells vs scrambled siRNA-transfected spheroids after 24 h of IFN-γ treatment (Figure 4)

To determine whether the increased invasion after BST2 silencing was not due to an increase in cell proliferation, BrdU cell proliferation assay was performed as described in *Materials and Methods*. No significant change in cell proliferation was observed in BST2 siRNA-transfected cells as compared to scrambled siRNA-transfected cells in the presence or absence of IFN-γ (Figure S1, Panel A).

### Effect of BST2 silencing on the expression of E-cadherin

**Figure 5.**
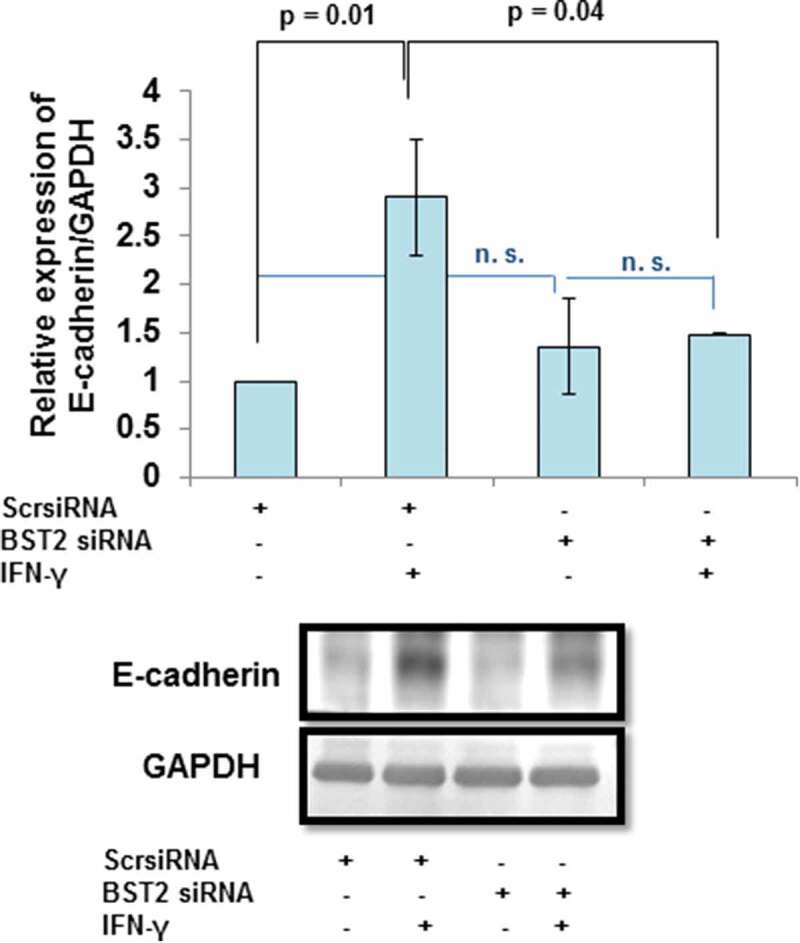
Expression of E-cadherin after BST2 silencing in HTR-8/SVneo cells treated with IFN-γ. HTR-8/SVneo cells (0.1 × 10^6^/well) were cultured overnight in 6-well culture plate at 37°C in 5% CO_2_ and 70% relative humidity and subsequently transfected with BST2 and scrambled siRNA. The scrambled siRNA- and BST2 siRNA-transfected cells were used to study the expression of E-cadherin by Western blotting using a mouse monoclonal antibody against E-cadherin as described in *Materials and Methods*. The bar graph shows the expression of E-cadherin at protein level in scrambled siRNA-transfected and BST2-silenced cells, respectively, on treatment with and without IFN-γ (10 ng/mL) and the representative blots of E-cadherin and GAPDH from one of the three experiments are appended. Each bar represents relative expression after normalization with GAPDH with respect to untreated scrambled siRNA-transfected cells. Values are expressed as mean ± SEM of three independent experiments.

Indirect immunofluorescence studies revealed that treatment of HTR-8/SVneo cells with IFN-γ (10 ng/mL) for 24 h leads to increased expression of E-cadherin as compared to untreated cells. Further, interaction of E-cadherin with actin was also observed (Figure 2, Panel b). To investigate if its expression is regulated by BST2, levels of E-cadherin were checked in BST2-silenced cells in the presence and absence of IFN-γ. The expression of E-cadherin was significantly (*p* = 0.01) increased in scrambled siRNA-transfected cells in the presence of IFN-γ vs untreated scrambled siRNA-transfected cells. Further, the expression of E-cadherin was significantly (*p* = 0.04) decreased in BST2 siRNA-transfected cells vs scrambled siRNA-transfected cells in the presence of IFN-γ (Figure 5). These findings suggest that the decrease in expression of E-cadherin after BST2 silencing may be responsible for increased invasion of HTR-8/SVneo cells.

### Effect of STAT1 silencing on the expression of BST2 and E-cadherin in HTR-8/SVneo cells after treatment with IFN-γ

**Figure 6.**
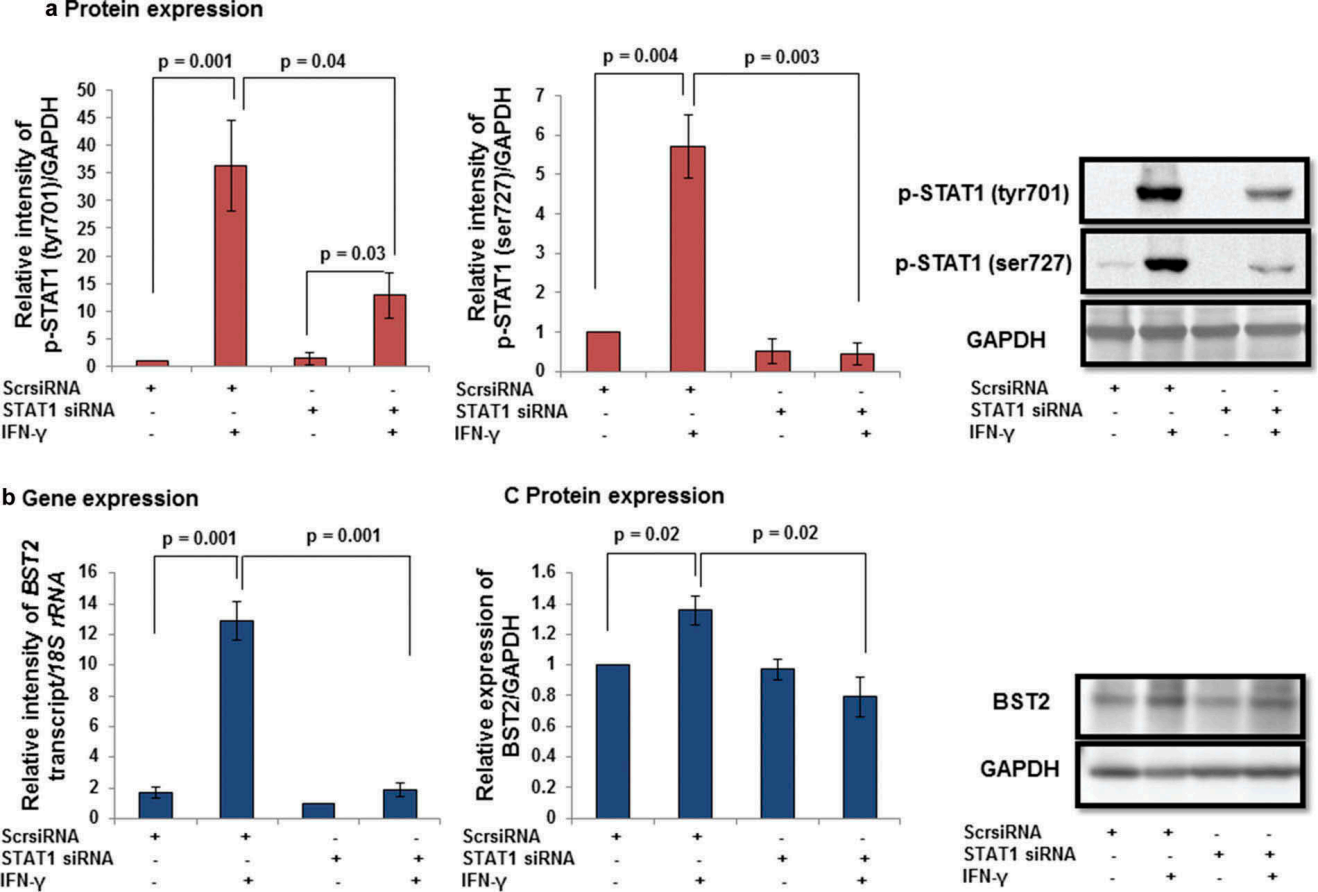
Expression of BST2 after STAT1 silencing in HTR-8/SVneo cells treated with IFN-γ. HTR-8/SVneo cells (0.1 × 10^6^/well) were seeded in 6-well cell culture plate and incubated at 37°C in 5% CO_2_ and 70% relative humidity and subsequently transfected with STAT1 and scrambled siRNA. Transfected cells were used to study the expression of p-STAT1 (tyr701) and p-STAT1 (ser727) in the presence and absence of IFN-γ (10 ng/mL) by Western blotting as described in *Materials and Methods*. Panel a shows the expression profile of p-STAT1 (tyr701) and p-STAT1 (ser727) in scrambled and STAT1 siRNA-transfected cells with and without IFN-γ treatment. The representative blots of the p-STAT1 (tyr701), p-STAT1 (ser727), and GAPDH are shown in Panel a. Panels b and c show the transcript and protein levels of BST2 in scrambled siRNA and STAT1 siRNA-transfected cells, respectively, on treatment with and without IFN-γ (10 ng/mL). The representative blots of BST2 and GAPDH from one of the three experiments are appended with Panel c. Each bar represents relative expression after normalization with either *18S rRNA* or GAPDH. Values are expressed as mean ± SEM of three independent experiments.

Considering the importance of STAT1 in IFN-γ-assisted decrease in invasion of HTR-8/SVneo cells from our previous report [9], the expression of BST2 and E-cadherin was checked at transcript and/or protein levels by qRT-PCR and Western blotting in STAT1-silenced cells. Initially, the status of p-STAT1 (tyr701) and p-STAT1 (ser727) in STAT1-silenced HTR-8/SVneo cells was studied by Western blotting. Expression of both p-STAT1 (tyr701) and p-STAT1 (ser727) was significantly increased in IFN-γ-treated scrambled siRNA-transfected cells with respect to untreated scrambled siRNA-transfected cells. In cells transfected with STAT1 siRNA, levels of p-STAT1 (tyr701) and p-STAT1 (ser727) were significantly reduced as compared to scrambled siRNA-transfected cells in the presence of IFN-γ (Figure 6, Panel a). Silencing of STAT1 also significantly (*p* = 0.001) downregulated the expression of *BST2* transcript with respect to scrambled siRNA-transfected cells treated with IFN-γ (Figure 6, Panel b). Similarly, the expression of BST2 was significantly downregulated at protein levels in STAT1-silenced cells vs scrambled siRNA-transfected cells treated with IFN-γ (Figure 6, Panel c). These results suggest that the expression of BST2 in HTR-8/SVneo cells is modulated by STAT1 in the presence of IFN-γ.

**Figure 7.**
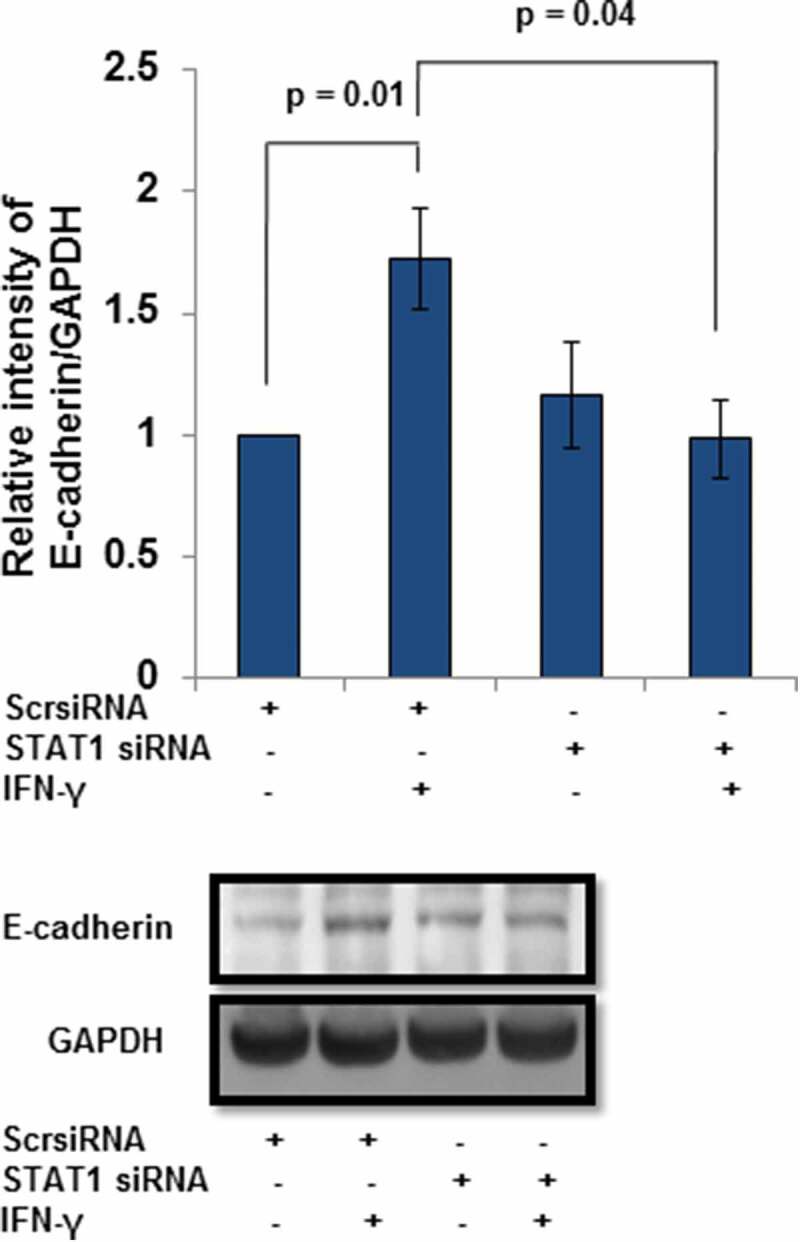
Expression of E-cadherin after STAT1 silencing in HTR-8/SVneo cells treated with IFN-γ. HTR-8/SVneo cells (0.1 × 10^6^/well) were grown overnight in 6-well culture plate at 37°C in 5% CO_2_ and 70% relative humidity. Subsequently, cells were transfected with STAT1 and scrambled siRNA and used to study the protein level of E-cadherin by Western blotting as described in *Materials and Methods*. The bar graph shows the expression of E-cadherin at the protein level in scrambled siRNA and STAT1 siRNA-transfected cells, respectively, on treatment with and without IFN-γ (10 ng/mL). Representative blots of E-cadherin and GAPDH from one of the three independent experiments are appended. Each bar represents the relative expression of E-cadherin after normalization with GAPDH with respect to untreated scrambled siRNA-transfected cells. Values are expressed as mean ± SEM of three independent experiments.

In addition to BST2, the expression of E-cadherin was also studied after STAT1 silencing. As expected, the protein levels of E-cadherin were significantly (*p* = 0.01) increased in scrambled siRNA-transfected cells treated with IFN-γ vs untreated scrambled siRNA-transfected HTR-8/SVneo cells (Figure 7). Interestingly, the expression of E-cadherin was also notably (*p* = 0.04) reduced in STAT1 siRNA-transfected cells compared to scrambled siRNA-transfected cells treated with IFN-γ (Figure 7).

### Role of AKT signaling pathway in IFN-γ-dependent decrease in invasion of HTR-8/SVneo cells

**Figure 8.**
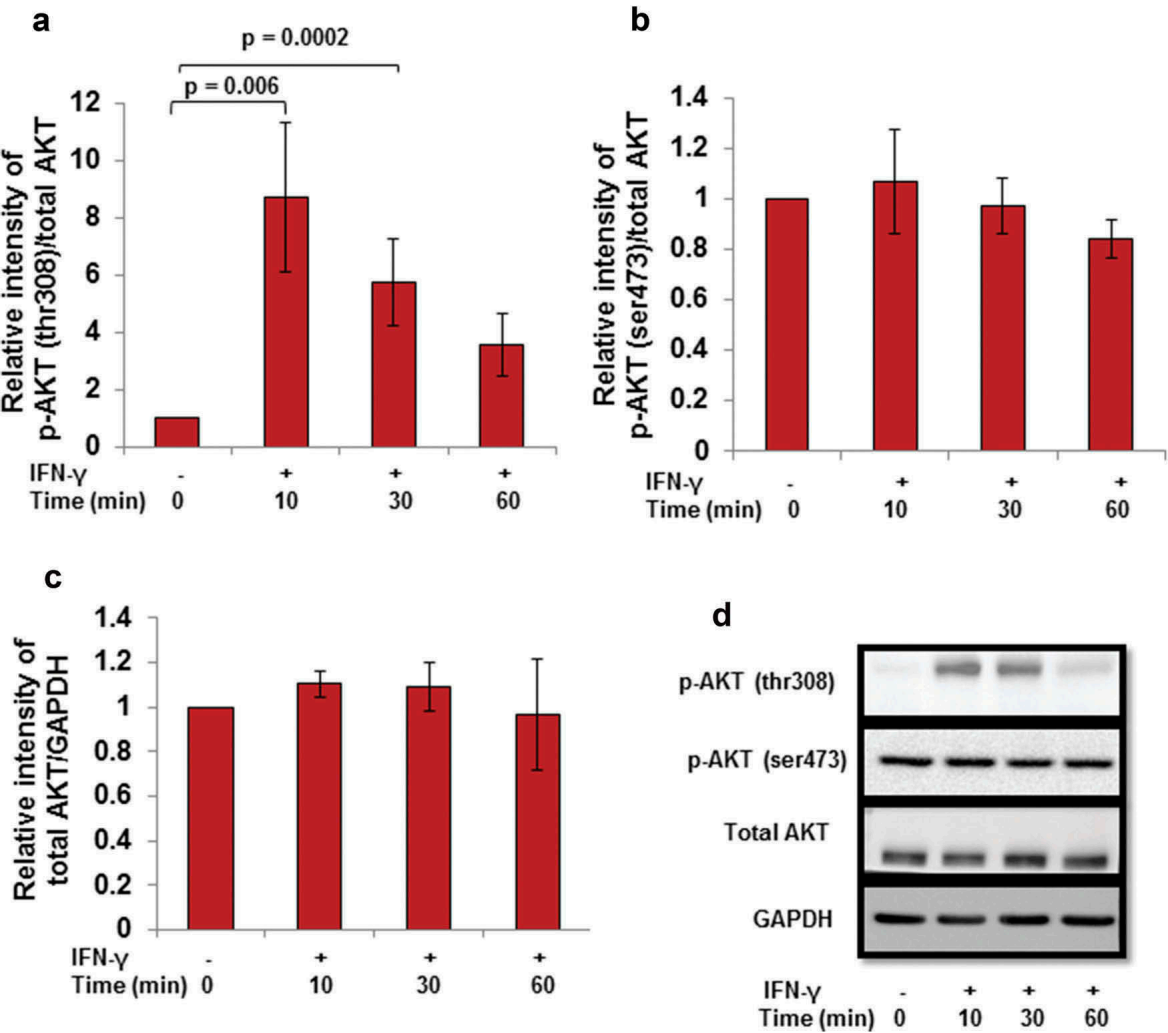
Effect of IFN-γ on the activation of AKT in HTR-8/SVneo cells. HTR-8/SVneo cells (0.1 × 10^6^/well) were cultured overnight in 6-well cell culture plate at 37°C in 5% CO_2_ and 70% relative humidity. The next day, cells were treated with IFN-γ (10 ng/mL) for 10, 30, and 60 min. After treatment, cell lysates were prepared and proteins resolved by 0.1% SDS–10% PAGE and processed for analysis of phosphorylated and total AKT by Western blotting as described in *Materials and Methods*. Panels a and b show the densitometric plot of p-AKT (thr308) and p-AKT (ser473) normalized with respect to total AKT and Panel c shows the densitometric plot of total AKT normalized with respect to GAPDH. Representative blots of p-AKT (thr308), p-AKT (ser473), total AKT, and GAPDH are appended as Panel d. The data are expressed as fold change with respect to 0-min control and values are shown as mean ± SEM of at least three independent experiments.

In addition to JAK/STAT signaling pathway, IFN-γ has been reported to activate the AKT signaling pathway [32,45]. However, the significance of AKT signaling pathway under the influence of IFN-γ in trophoblast invasion is not known. Cells were treated with IFN-γ for 0, 10, 30, and 60 min; cell lysates were made and proteins resolved by 0.1% SDS–10% PAGE for Western blotting. Treatment of cells with IFN-γ significantly activated p-AKT (thr308) at 10 min (p = 0.006) and 30 min (p = 0.0002), with respect to 0-min time point (Figure 8, Panels a and d). However, no significant changes in the expression of p-AKT (ser473) were observed at any of the time points studied as compared to 0 min control (Figure 8, Panels b and d). No significant changes in the expression of total AKT were also observed (Figure 8, Panels c and d).

**Figure 9.**
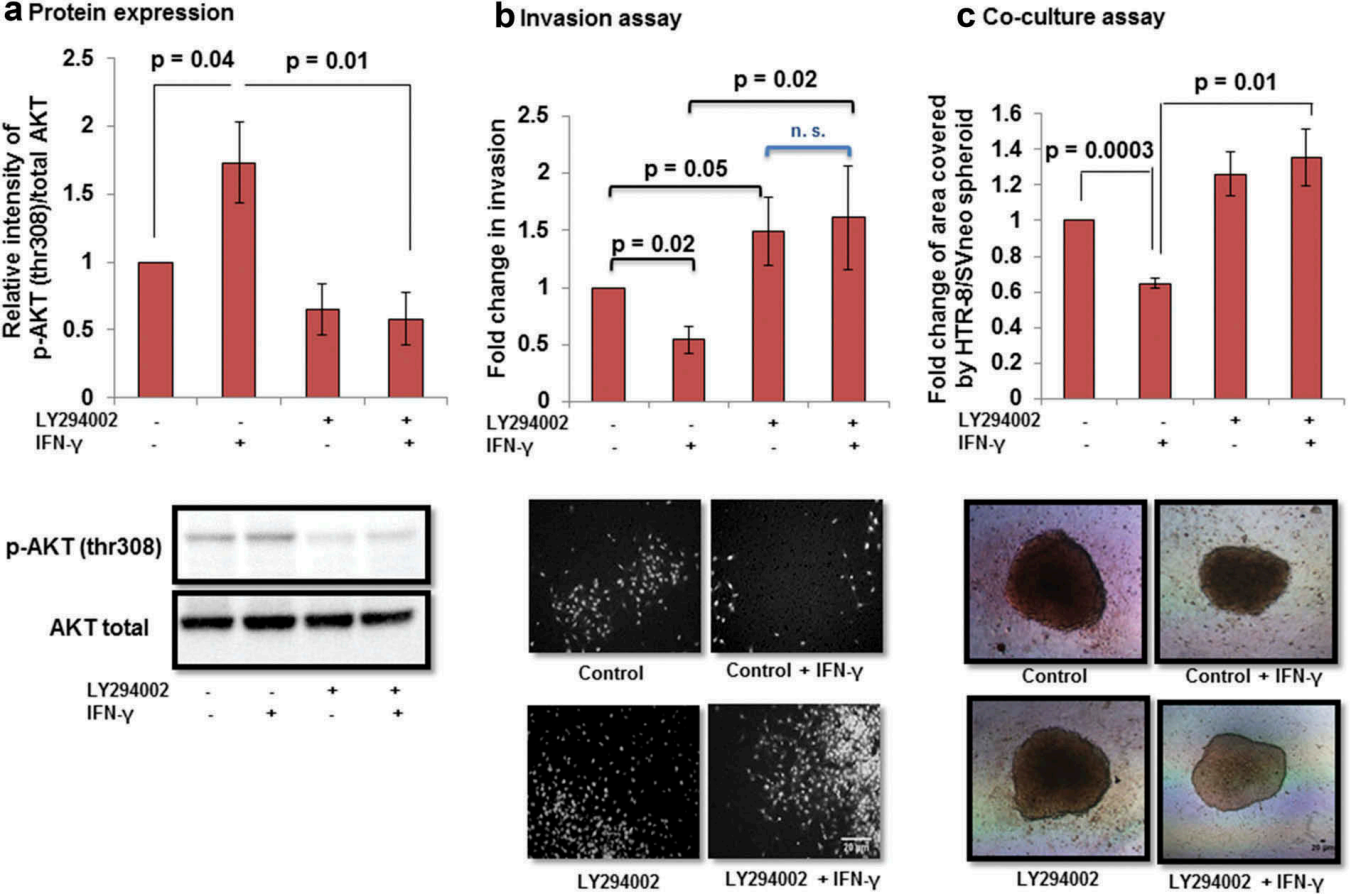
Effect of AKT inhibition by PI3K inhibitor on the invasion of HTR-8/SVneo cells treated with IFN-γ. To study the importance of AKT signaling pathway, HTR-8/SVneo cells (0.1 × 10^6^/well) were cultured in 6-well cell culture plate overnight at 37°C in 5% CO_2_ and 70% relative humidity. The next day, cells were either pretreated or not pretreated with PI3K inhibitor (LY294002, 50 μM) for 2 h. Subsequently, cells were used for either Western blotting to check the expression of p-AKT (thr308) and total AKT or invasion assay in the presence and absence of IFN-γ (10 ng/mL) for 24 h. Panel a shows the densitometric plot of p-AKT (thr308) normalized with respect to total AKT. Representative blot of p-AKT (thr308) and total AKT has been appended along with Panel a. Panel b shows the fold change in the invasion of control and LY294002 pretreated cells on subsequent treatment with and without IFN-γ for 24 h. Representative images of the cells in the invasion assay has been included in Panel b. The results are expressed as mean ± SEM of fold change in the invasion with respect to control HTR-8/SVneo cells without pretreatment with LY294002 and IFN-γ treatment, observed in three independent experiments. Panel c shows fold change in the area of spreading of control HTR-8/SVneo spheroids and LY294002 pre-treated HTR-8/SVneo spheroids, respectively, with/without IFN-γ treatment for 24 h, on the monolayer of Ishikawa cells with respect to untreated control. The representative images of the spheroids are appended with Panel c. Scale bar represents 20 μm.

Further, the importance of the AKT signaling pathway in IFN-γ-assisted decrease in invasion of HTR-8/SVneo cells was validated by using PI3K chemical inhibitor LY294002 [2-(4-morpholinyl)-8-phenyl-chromone]. Cells were pretreated with LY294002 (50 μM) for 2 h. No significant changes in the cell viability were observed in LY294002 pretreated cells and also in those which were subsequently treated with IFN-γ for 24 h (Figure S2). Pretreatment of cells with LY294002 followed by IFN-γ treatment led to a significant inhibition in the phosphorylation of p-AKT (thr308) at 10, 30, and 60 min *vs* LY294002 untreated cells but subsequently treated with IFN-γ (Figure S3). Moreover, pretreatment of HTR-8/SVneo cells with LY294002 also led to a significant reduction in the IFN-γ-mediated increased expression of p-STAT1 (tyr701) as well as p-STAT1 (ser727) (Figure S4). Expression of p-AKT (thr308) was also significantly higher in IFN-γ-treated vs control cells at 24 h (Figure 9, Panel a). Pretreatment of cells with LY294002 for 2 h showed significant (*p* = 0.01) reduction in the expression of p-AKT (thr308) as compared to control cells in the presence of IFN-γ (Figure 9, Panel a). Further, LY294002 pretreated cells were utilized for matrigel matrix invasion assay with and without treatment of IFN-γ. A significant (*p* = 0.02) decrease in the number of invading cells was observed in IFN-γ-treated cells vs control cells without pretreatment with LY294002. Inhibition of AKT signaling pathway by LY294002 led to a remarkable (~1.5 fold; *p* = 0.05) increase in the number of invading cells without any subsequent treatment with IFN-γ *vs* LY294002/IFN-γ untreated control cells. Similarly, a significantly higher number of invading cells (~3.2 fold; *p* = 0.02) were also observed in LY294002 pretreated cells and further treated with IFN-γ as compared to cells not pretreated with LY294002 and subsequently treated with IFN-γ (Figure 9, Panel b). Subsequently, co-culture of LY294002 pretreated HTR-8/SVneo spheroids grown on the monolayer of Ishikawa cells showed a significant increase in the spreading compared to control HTR-8/SVneo spheroids after 24 h of IFN-γ treatment (Figure 9, Panel c).

To rule out the possibility that the increased invasion after pretreatment of LY294002 was not due to an increase in cell proliferation rate, LY294002 pretreated cells were used for BrdU cell proliferation assay as described in *Material and Methods*. No significant changes in proliferation were observed in LY294002 pretreated cells as compared to control cells treated with and without IFN-γ (Figure S1, Panel B).

### Relevance of AKT signaling pathway on the expression of BST2 and E-cadherin

**Figure 10.**
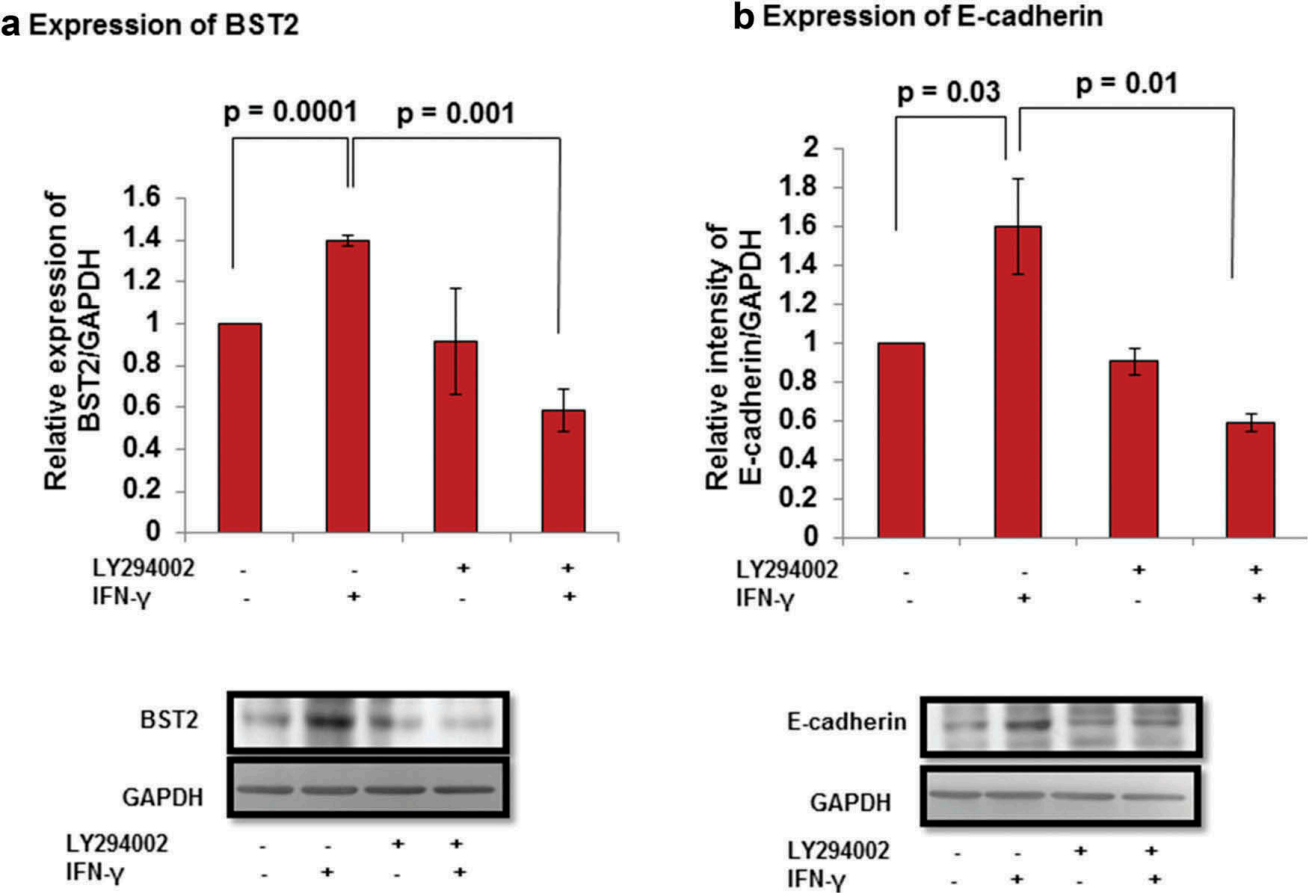
Expression of BST2 and E-cadherin after AKT inhibition in HTR-8/SVneo cells treated with IFN-γ. HTR-8/SVneo cells (0.1 × 10^6^/well) were cultured overnight in 6-well cell culture plate at 37°C in 5% CO_2_ and 70% relative humidity. The next day, cells were pretreated in the presence and absence of LY294002 (50 μM) for 2 h followed by treatment with IFN-γ (10 ng/mL) for 24 h and used to study the expression of BST2 and E-cadherin by Western blotting as described in *Materials and Methods*. Panel a shows the bar graph of BST2 at the protein level in untreated and pretreated LY294002 cells, respectively, on treatment with and without IFN-γ. Panel b shows the expression of E-cadherin at the protein level in untreated and LY294002 pretreated cells, respectively, on subsequent treatment with and without IFN-γ. Each bar represents relative expression after normalization with GAPDH with respect to LY294002 untreated control cells and without treatment of IFN-γ. Values are expressed as mean ± SEM of three independent experiments. Representative blots of BST2, E-cadherin, and GAPDH from one of the three independent experiments are appended with respective panels.

In order to determine if activation of AKT signaling pathway by IFN-γ in HTR-8/SVneo cells has any role in the levels of BST2 and E-cadherin, cells were treated with and without IFN-γ for 24 h, which were either pretreated or not with LY294002 for 2 h. After 24 h of IFN-γ treatment, cells were subsequently processed for Western blotting to determine the expression of BST2 and E-cadherin. In cells that were not pretreated with LY294002, a significant (*p* = 0.0001) upregulation in the expression of BST2 was observed in IFN-γ-treated cells vs IFN-γ untreated control cells (Figure 10, Panel a). However, the expression of BST2 was significantly (*p* = 0.001) downregulated in LY294002 pretreated cells on subsequent treatment with IFN-γ vs control cells subsequently treated with IFN-γ (Figure 10, Panel a).

Furthermore, the expression of E-cadherin was significantly increased (*p* = 0.03) in IFN-γ-treated cells vs untreated cells in the absence of pretreatment with LY294002 (Figure 10, Panel b). However, a significant (*p* = 0.01) decrease in the expression of E-cadherin in LY294002 pretreated cells followed by IFN-γtreatment was observed vs IFN-γ-treated control cells (Figure 10, Panel b).

## Discussion

Understanding the molecular mechanism of trophoblast invasion is important for successful pregnancy. The molecular basis of IFN-γ-mediated trophoblast invasion is not yet well established, although its action as an inhibitor of invasion has been reported by few studies [8,9]. Here, HTR-8/SVneo cells were used as a model to study trophoblast invasion. This cell line has been extensively used by several investigators to delineate the mechanism of trophoblast invasion as it shows resemblance with human first-trimester EVTs [46–48].

**Figure 11.**
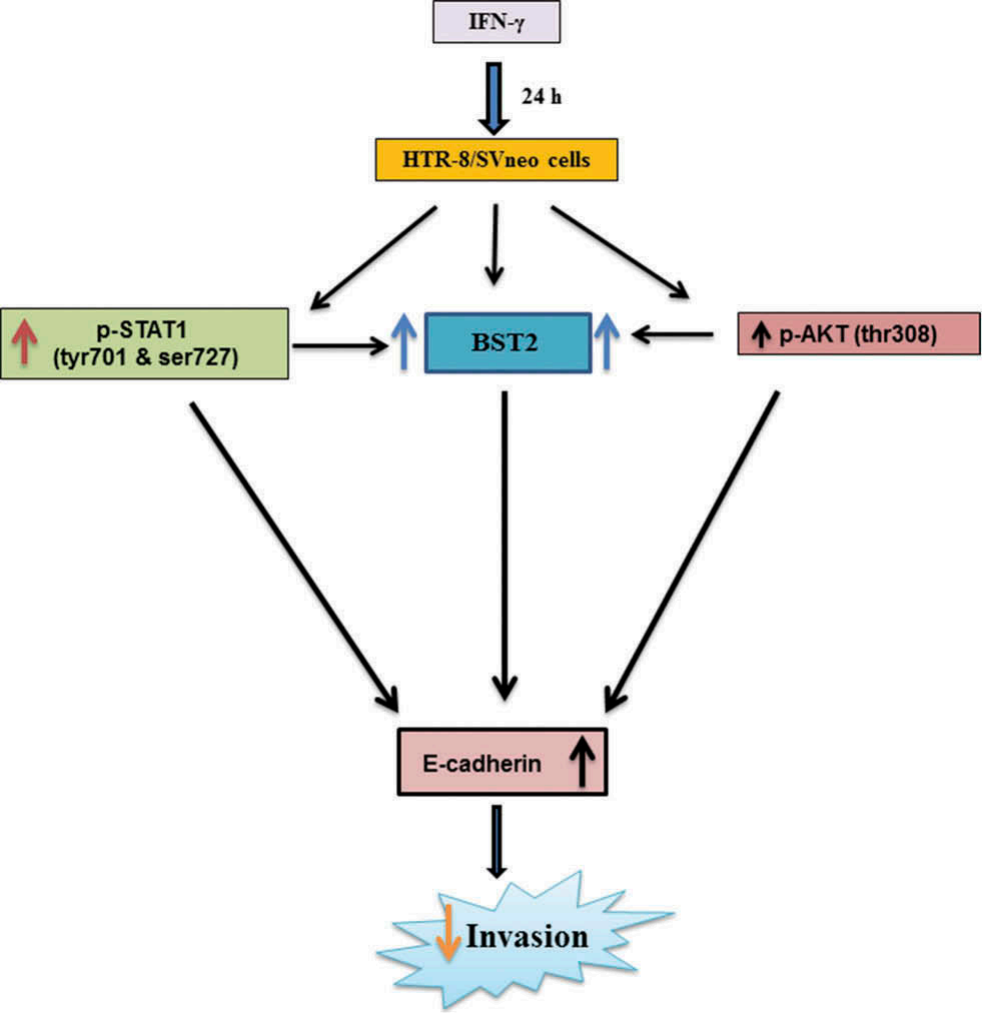
Role of BST2, E-cadherin, and signaling pathways in IFN-γ-mediated decrease in invasion of HTR-8/SVneo cells. Treatment of HTR-8/SVneo cells with IFN-γ leads to upregulation in the expression of BST2 and concomitantly activates JAK/STAT and AKT signaling pathways. Silencing or inhibition of BST2 and AKT signaling pathway abrogates the effect of IFN-γ on the decreased invasion of HTR-8/SVneo cells. Further, silencing of STAT1 and inhibition of AKT signaling pathways also inhibit the expression of BST2. Interestingly, the expression of E-cadherin was regulated by STAT1 and AKT signaling pathways and also by levels of BST2, which suggest their role in IFN-γ-mediated decrease in invasion of HTR-8/SVneo cells.

In our previous study, next-generation sequencing revealed an increased expression of BST2 in HTR-8/SVneo cells treated with IFN-γ for 24 h [9]. In the present study, upregulation in the expression of BST2 in HTR-8/SVneo cells after IFN-γ treatment was further confirmed at the transcript and protein level (Figure 1, Panels a and b, Figure 11), which supports the published literature that BST2 is an IFN-inducible protein [16]. The overexpression of BST2 has been associated with increased metastasis in MDA-231BO in comparison to the primary human nonmetastatic MDA-231 breast cancer cell line. These studies demonstrated the role of BST2 in cancer cell migration and proliferation [24,49]. Moreover, BST2 also promotes invasion and migration of tamoxifen-resistant breast cancer cells MCF-7 via TCF-7 [31]. In contrast to these studies, BST2 is also known to inhibit the cell growth and motility by inhibiting the activation of MMP2, which is a key enzyme involved in invasion and migration of cells [26]. In our study, inhibition of BST2 level led to an increase in the invasion of BST2-silenced cells vs scrambled siRNA-transfected cells with and without treatment of IFN-γ (Figure 3, Panel c, Figure 11). In addition to matrigel matrix invasion assay, we have also observed increased area of spreading of BST2-silenced cells from HTR-8/SVneo spheroids as compared to scrambled siRNA-transfected spheroids on the monolayer of Ishikawa cells in the presence of IFN-γ (Figure 4). These observations suggest that BST2 could act as a promoter or inhibitor of invasion/spreading depending on the cell type and environment. Further, we studied the effector proteins associated with BST2 which regulate invasion of trophoblast cells under the influence of IFN-γ. Studies showed that the expression of E-cadherin promotes cohesive cell morphology and decreases cell motility and invasion of intermediate trophoblast cells [43,50]. Here, we observed an increased expression of E-cadherin in scrambled siRNA-transfected cells after treatment with IFN-γ than untreated control. Subsequently, the expression of E-cadherin was significantly down-regulated in BST2-silenced cells with respect to scrambled siRNA-transfected cells after IFN-γ treatment (Figure 5). In literature, BST2 is known to interact with other proteins with its cytoplasmic tail and alter the growth and motility of cells [21,26]. Moreover, the cytoplasmic tail of the BST2 also interacts with the actin cytoskeleton with the help of Rho GAP interacting with CIP4 homolog protein 2 (RICH2; [22]). Similarly, E-cadherin is also known to modify actin cytoskeleton by interaction with catenin protein complex. The interaction between E-cadherin and actin provides adhesive state to cells which help in gathering of cells together [51]. Here, we have also shown the interaction of BST2 and E-cadherin with actin as determined by immunofluorescence, which suggests that BST2 and E-cadherin protein interact with actin cytoskeleton and helps in invasion of these cells (Figure 2, Panels a and b). So far, the connection between BST2 and E-cadherin has not been shown by any study. Our studies suggest for the first time that BST2 might be regulating E-cadherin expression, and these proteins further regulate the invasive ability of HTR-8/SVneo cells in the presence of IFN-γ.

As activation of STAT1 plays an important role in IFN-γ-mediated decrease in invasion [9], its function with respect to regulation of effector proteins like BST2 and E-cadherin was also studied. Studies have shown that BST2 is activated by the addition of IFNs and also regulated by STAT1 [16,52]. Here, we observed a decrease in the expression of BST2 both at transcript and protein levels in STAT1-silenced cells compared to scrambled siRNA cells after treatment with IFN-γ, which is well supported by previous studies (Figure 6, Panels b and c, Figure 11). Studies showed that the level of E-cadherin is downregulated by STAT3 activation after the addition of oncostatin M (OSM), which further stimulates invasion of HTR-8/SVneo cells [53]. In addition, activation of STAT1 and STAT3 has an opposite effect on tumor survival and growth by regulating a plethora of effector proteins [54]. In this study, silencing of STAT1 led to inhibition in the expression of E-cadherin, which suggests that an increase in the invasion of cells could be due to the downregulation of E-cadherin (Figure 7 and 11).

Activation of AKT under the influence of different cytokines and growth factors has been shown to promote trophoblast invasion [36–39]. However, there are few studies that suggest that AKT also inhibits tumor motility, invasion, and metastasis in the case of breast cancer [40,55]. In this context, there is no information on the role of AKT in IFN-γ-mediated invasion of trophoblast cells. Treatment of cells with IFN-γ led to phosphorylation of AKT at thr308 residue at various time points (10, 30, and 60 min) studied; however, no significant changes were observed in the phosphorylated form of AKT at ser473 residue (Figure 8, Panels a, b, and d, Figure 11). It has been documented previously that p-AKT (thr308) leads to maximal activation of AKT, whereas activation at p-AKT (ser473) only indicates partial activation [56]. Further, inhibition of AKT by pretreatment of HTR-8/SVneo cells with PI3K inhibitor (LY294002) led to a significant increase in the number of invading cells after IFN-γ treatment without affecting proliferation rate and cell viability (Figure 9, Panel band Figures S1 and S2). In addition, co-culture of LY294002 pretreated HTR-8/SVneo spheroids also showed an increased area of spreading on the monolayer of Ishikawa cells in the presence of IFN-γ (Figure 9, Panel c). Increase in the number of invaded cells/spreading after inhibition of AKT signaling pathway suggests that AKT regulates IFN-γ-mediated invasion of HTR-8/SVneo cells. However, our previous report also implies that STAT1 signaling pathway is involved in IFN-γ-mediated invasion of these cells [9]. Previously published reports performed in different tissue samples and cell lines suggest that AKT regulates JAK/STAT signaling pathway and control various biological processes [32,57,58]. We have also checked the effect of LY294002 on the phosphorylation status of STAT1 (tyr701) and STAT1 (ser727) residues. Interestingly, we found that the phosphorylation of STAT1 (tyr701 and ser727) was significantly reduced in LY294002 pretreated cells as compared to LY294002 untreated cells subsequent to treatment with IFN-γ (Figure S4).

Subsequently, the role of effector proteins which are regulated by AKT signaling pathway to control the IFN-γ-dependent decrease in invasion of cells has also been studied. Studies showed that silencing of BST2 with siRNA inhibits the phosphorylation of AKT and ERK1/2 in bladder cancer cells [28]. Interestingly, in our study, inhibition of AKT signaling pathway in cells downregulate the expression of BST2 after IFN-γ treatment (Figure 10, Panel a; Figure 11). This suggests that the expression of BST2 is under the regulation of both STAT1 and AKT, and these signaling pathways are responsible for IFN-γ-dependent decrease in invasion of cells. There are several reports which emphasized that AKT signaling pathway inhibits the level of E-cadherin and promotes EMT in different cancer cells [59–61]. Also the presence of E-cadherin on the membrane brings the signal from outside to inside, leading to activation of PI3K/AKT signaling pathway that regulates the formation of cell–cell junction of epithelial cells and proliferation of epithelial ovarian cancer cells [62,63]. In the present study, decreased expression of the E-cadherin was observed after inhibition of AKT signaling pathway in cells subsequent to treatment with IFN-γ (Figure 10, Panel b; Figure 11). These observations suggest that AKT signaling pathway could be activated by different cytokines acting on the same effector molecule, but the action may differ that depends on the cell type.

In summary, the studies presented herein showed upregulation in the expression of BST2 in HTR-8/SVneo cells after IFN-γ treatment and its silencing promotes their invasion/spreading by downregulating expression of E-cadherin. Silencing of STAT1 by siRNA as well as inhibition of the activation of AKT by inhibitor led to a decrease in the expression of BST2 and E-cadherin, thereby reversing the observed decreased invasion/spreading of HTR-8/SVneo cells by IFN-γ, suggesting their relevance in IFN-γ-mediated invasion of trophoblast cells.

## Materials and methods

### In vitro *culture of HTR-8/SVneo cells and Ishikawa cells*

HTR-8/SVneo cells derived from human first-trimester placental explant cultures and immortalized by Simian virus (SV40) large T antigen [46] were cultured in 1:1 ratio of Dulbecco’s modified Eagle medium (DMEM, Sigma-Aldrich Inc., St. Louis, MO, USA; #D1152-10L) and Ham’s F-12 medium (Sigma-Aldrich Inc., #N3520-10L) supplemented with 10% fetal bovine serum (FBS; Gibco® Life Technologies, Grand Island, NY, USA; #10270) and antibiotic-antimycotic cocktail [streptomycin (100 units/mL), penicillin (100 µg/mL), and amphotericin B (0.25 µg/mL; MP Biomedicals, Santa Ana, CA, USA; #1674049)] at 37°C under 5% CO_2_ and 70% relative humidity. Ishikawa endometrial epithelial cells were also cultured as described for HTR-8/SVneo cells. HTR-8/SVneo cells were treated with geneticin sulfate (G418; Himedia, Kelton, PA, USA; #TC025) at 75 μg/mL after every third passage to inhibit the growth of untransfected HTR-8/SVneo cells.

### Invasion assay

Matrigel matrix invasion assay was carried out as reported previously [9]. In brief, 50 μL of growth factor reduced matrigel matrix (1 μg/mL, BD Bioscience, San Jose, CA, USA; #356230) was coated in each transwell insert with 8-μm filter pore size (Greiner Bio-One, Kremsmunster, Austria; #662638) and placed in 24-well plate for overnight at 37°C in CO_2_ incubator. After overnight incubation, 300 μL of medium supplemented with 1% FBS with and without optimized concentration of IFN-γ (10 ng/mL; Peprotech, Rocky Hill, NJ, USA #30002) was added into the lower well. Further, naïve or treated HTR-8/SVneo cells as per experimental design (0.1 × 10^6^ cells/150 μL) were added into the upper well of transwell insert in the presence or absence of IFN-γ and kept for 24 h at 37°C with 5% CO_2_ and 70% relative humidity. After 24-h incubation, noninvading cells, medium, and matrigel were removed carefully from the upper chamber of transwell insert with a moist cotton swab. The lower side of the transwell insert having cells was fixed using chilled methanol at 4°C for 10 min followed by washing with 50 mM PBS (pH-7.4) and staining with Hoechst nuclear staining dye (0.2 μM; Thermo Fisher Scientific, Waltham, MA, USA; #H3570) for 10 min at 37°C. After staining, the membrane of the transwell insert was cut and mounted with immersion oil on the slide facing the lower surface of the membrane to the upper side. The cells which were present on the lower side of the membrane were counted using a fluorescent phase-contrast microscope (Nikon Instruments Inc., Melville, NY, USA), and pictures of the invaded cells were taken with the help of Image Pro-plus software. The fold change in invasion was calculated by dividing the number of cells on the membrane of IFN-γ treated with the number of the cells present on the untreated membrane.

### Inhibition of AKT signaling by PI3K inhibitor (LY294002)

HTR-8/SVneo cells (0.1 × 10^6^/well) were plated into six-well cell culture plate in medium with 10% FBS and incubated overnight at 37ºC with 5% CO_2_ and 70% relative humidity. The next day, cells were deprived of serum for 4 h by incubating in plain medium without FBS. Subsequently, cells were pre-treated with PI3K inhibitor LY294002 (50 μM; Cell Signaling Technology Inc., Danvers, MA, USA; #9901) for 2 h to inhibit the phosphorylation of AKT. After 2 h, cells were used for invasion assay, cell viability assay, and cell proliferation assay with or without IFN-γ treatment. In addition, cells were also processed for Western blotting at 0, 10, 30, and 60 min after IFN-γ treatment to study the activation of signaling pathways. Further, cell lysates were also used for Western blotting after 24 h treatment with and without IFN-γ to study the changes in the expression of various effector proteins.

### Cell viability assay

HTR-8/SVneo cells were cultured overnight followed by serum starvation for 4 h as described earlier. Subsequently, cells were pretreated with and without LY294002 for 2 h in a plain medium. After pretreatment with and without LY294002, cells were further treated with and without IFN-γ. After treatment, cells were trypsinized using 0.5% trypsin + 0.2% EDTA, mixed in physiological saline (0.9% NaCl), and stained with 5 μM SYTOX^TM^ green (Thermo Fisher Scientific, #S7020). Using flow cytometry, the percentage of SYTOX green-stained cells (dead cells) was estimated at 488-nm excitation.

### Gene silencing by siRNA

HTR-8/SVneo cells (0.1 × 10^6^) suspended in medium containing 10% FBS were seeded in a six-well cell culture plate and grown at 37°C in 5% CO_2_ and 70% relative humidity. The next day, cells were transfected with an optimized concentration of scrambled siRNA, STAT1 siRNA, and BST2 siRNA (40 pmole; Santa cruz Biotechnology Inc., Santa Cruz, CA, USA; #sc-37007, #sc-4123, and #sc-60294, respectively), using lipofectamine 3000 (Life Technologies, #L3000-015) and Opti-MEM® medium (Gibco®, #31985070). Briefly, cells were rinsed with Opti-MEM® medium, and fresh Opti-MEM® (750 µL) medium was added to each well of six-well plate. Further, the optimized concentration of siRNA along with Opti-MEM® medium was mixed and volume made up to 125 µL. In a different eppendorf tube, 7 µL of lipofectamine 3000 was mixed with Opti-MEM® medium to make a total volume of 125 µL and kept for 5 min at room temperature (RT). After 5 min, both the solutions were mixed and kept for 10 min at RT. After incubation, the solution was added dropwise in the respective wells. After 6 h of incubation, medium supplemented with 10% FBS was added to the respective wells. After transfection for 48 h, cells were processed for Western blotting, qRT-PCR, and various functional assays.

### Cell proliferation assay

Cell proliferation assay was performed using 5-bromo-2ʹ-deoxy-uridine (BrdU) cell proliferation assay kit as per the manufacturer’s instructions (Merck Millipore, Burlington, MA, USA; #2750). HTR-8/SVneo cells transfected with either scrambled siRNA or BST2 siRNA were trypsinized, counted, and 8000 cells/well were seeded in 96-well cell culture plates in 200-μL plain medium with 20-μL BrdU reagent (1:500 dilution) with and without IFN-γ and kept at 37°C with 5% CO_2_ and 70% relative humidity for 24 h. In another experiment, HTR-8/SVneo cells pretreated with or without LY294002 and subsequently treated with IFN-γ as described earlier were also processed for BrdU incorporation. After 24-h incubation, the medium was aspirated, and cells were fixed using fixing solution and incubated with prediluted BrdU-detection antibody (100 μL/well) for 1 h at RT. Further, cells were washed with wash buffer provided in the kit. Subsequently, cells were incubated with peroxidase-conjugated goat anti-mouse IgG (1:2000) for 30 min at RT followed by three times washing with wash buffer. After washing, cells were incubated with TMB (3, 3ʹ, 5, 5ʹ-tetramethylbenzidine) peroxidase substrate solution for 30 min in the dark. Further, stop solution was added in each well to stop the reaction and absorbance determined at 450/550 nm using a microplate reader (BioTek Instruments, Winooski, VT, USA). Percent cell proliferation was determined by normalized absorbance (absorbance in experimental group-absorbance of blank wells) of untreated group by treated group multiplied by 100.

### Co-culture of HTR-8/SVneo spheroids with Ishikawa cells

HTR-8/SVneo spheroids were prepared by hanging drop method as described previously [64,65].

Briefly, 2500 naïve HTR-8/SVneo cells/20 μL drop in DMEM + Ham’s F12 medium supplemented with 10% FBS were plated on the lid of a petri dish and the lower part of the petri dish filled with sterile PBS. The lid was inverted slowly on the PBS-filled petri dish and incubated at 37ºC with 5% CO_2_ and 70% relative humidity for 48 h. Similarly, spheroids of HTR-8/SVneo cells silenced for BST2 along with appropriate control were also made. In one of the experimental setup, spheroids were pretreated with and without LY294002 (50 μM) for 2 h. Further, spheroids were stained with cell tracking dye (1:300 dilution, CM Dil; Life technologies; #C7000) for 30 min. Subsequently, spheroids were plated on the top of monolayer of Ishikawa cells in the presence or absence of IFN-γ in 10% FBS medium. Following 24-h co-incubation, images were taken and the area of spread by cells from spheroid was measured by ImageJ software (http://rsb.info.nih.gov/ij/).

### Quantitative real-time polymerase chain reaction (qRT-PCR)

HTR-8/SVneo cells (0.1 × 10^6^/well) were seeded in six-well plates and incubated for 24 h at 37ºC in 5% CO_2_ and 70% relative humidity. The next day, cells were serum starved at least for 4 h by incubating cells in plain medium without FBS before treatment with IFN-γ for 24 h and employed to study relative levels of gene expression. After treatment, total RNA was extracted using RiboZol™ (AMRESCO®, Solon, OH, USA; #N580) according to the manufacturer’s instructions. Isolated RNA was analyzed by NanoDrop 3300 spectrophotometer (Thermo Scientific, NanoDrop Products, DE, USA) for its quantitation and purity and was used to prepare complementary DNA (cDNA).

To prepare cDNA, 5 μg of RNA was taken in an Eppendorf tube followed by addition of dNTP mix, Oligo (dT)_18_ primer, random hexamer primer (Fermentas International Inc., Burlington, ON, Canada; #18427088, #SO132, #SO142, respectively), and the total volume was made up to 14.5 μL and kept at 65°C for 5 min followed by 5-min cooling at 4°C. Further, Maxima RT enzyme (Fermentas International Inc., #EP0742), RT buffer, and RiboLock RNase Inhibitor (Fermentas International Inc., #EO0381) were added into each tube and mixed well. The total volume was made up to 20 μL and incubated for 10 min at 25ºC and 30 min at 50ºC. The reaction was stopped by heating at 85ºC for 5 min.

The qRT-PCR reactions for BST2 (forward primer: 5ʹ-AGCGACTGAGAAGAGAAAACCA-3ʹ, reverse primer: 5ʹ-TGTTCAAGCGAAAAGCCGAG-3ʹ) were performed in duplicates in a total volume of 20 µL for the analysis of its expression profile. The reaction was carried out containing SYBR Premix Ex Taq II (Tli RNaseH Plus) master mix (Takara Bio Inc., Kusatsu, Shiga, Japan; #RR820A), synthesized cDNA (diluted 10 times), and gene-specific primers (0.1 µM) in Stratagene Mx3005P (Agilent Technologies Inc., Santa Clara, CA, USA). The temperature profile for target gene amplification was as described in our previous report [9]. A single peak in the dissociation curve analysis validated gene-specific amplification. *18S rRNA* (forward primer: 5′-GGAGAGGGAGCCTGAGAAAC-3′, reverse primer: 5′ CCTCCAATGGATCCTCGTTA 3′) was run in parallel to normalize the average threshold cycle (Ct) values. These relative ΔCt values were used to determine the fold change in the expression (relative expression) between treated and untreated control groups.

### Western blot analysis

HTR-8/SVneo cells (0.1 × 10^6^/well) were grown for 24 h in 6-well plate in medium supplemented with 10% FBS at 37°C in 5% CO_2_ and 70% relative humidity. Before treatment, cells were serum-starved for at least 4 h. Further, cells were treated with IFN-γ for 10, 30, and 60 min and 24 h. After incubation, the medium was removed and cells were lyzed by freezing in liquid nitrogen followed by thawing in cell lysis buffer (100 μL) along with complete phosphatase and protease inhibitor cocktail (Roche Diagnostic, Mannheim, Germany; #4906845001 and #05892791001) as previously described [9]. Subsequently, cell lysates were spinned at 10,000 × *g* at 4°C for 10 min, supernatant collected and stored at −20ºC till used.

The protein concentration in cell lysates was determined using BSA as standard by Bicinchoninic Acid (Pierce, Waltham, MA, USA; #23225) colorimetric assay as per manufacturer’s instructions. Cell lysate (40-μg/lane) was loaded in 0.1% SDS–10% PAGE and proteins resolved at 25 mA for 1–1.5 h. The resolved proteins were transferred onto the 0.45-μm nitrocellulose membrane (mdi Membrane Technologies, Ambala Cantt., Haryana, India; #SCNJ8102XXXX101). After the transfer of the proteins, the nitrocellulose membrane was blocked using 5% BSA in PBS for 1 h at RT. After blocking, the membrane was washed with PBS for 5 min. Further, the individual blots were kept overnight at 4°C with 1:1000 dilution of rabbit monoclonal antibodies against p-AKT (thr308), p-AKT (ser473), total AKT, p-STAT1 (tyr701), p-STAT1 (ser727) (Cell Signaling Technology Inc., #13038, #4060, #4691, #9167, #9177) and 1:400 dilution of mouse monoclonal antibodies against BST2 (Santa Cruz Biotechnology Inc., #sc-310719), 1:10,000 dilution of GAPDH antibody (Abgenex, Bhubaneswar, Odisha, India; #10-10011), and 1:1000 dilution of E-cadherin (Cloud-Clone Corp., Houston, TX, USA; #MAA017Hu22) in 5% BSA in 50 mM tris-buffered saline pH-7.4 supplemented with 0.1% Tween 20 (TBST). After subsequent washing with TBST, the membrane was further incubated with 1:2000 dilution of HRP conjugated goat anti-rabbit or anti-mouse IgG antibody in TBST with 5% BSA for 1 h at RT. After incubation in secondary antibody, blots were again washed thrice with TBST and developed using Immobilon chemiluminescent substrate (Merck Millipore, #WBKLS0500) and photographs were obtained by FluorChem E system (Protein Simple, San Jose, CA, USA). The intensity of bands was quantified using ImageJ software (http://rsb.info. nih.gov/ij/).

### Indirect immunofluorescence

HTR-8/SVneo cells (20,000 cells/well) were seeded on the coverslips in 24-well cell culture plate and incubated overnight at 37°C in 5% CO_2_ and 70% relative humidity. The next day, cells were incubated in plain medium for 4 h followed by treatment with IFN-γ in a plain medium for 24 h. After treatment, cells were fixed using 4% paraformaldehyde at RT for 10 min. After fixing, cells were washed 2 times with PBS followed by blocking in 3% BSA in PBS for 1 h at RT. Further, cells were incubated with 1:50 dilution of primary antibody against either BST2 (Santa Cruz Biotechnology Inc., # sc-390719) or E-cadherin (Cloud-Clone Corp., #MAA017Hu22) in 3% BSA in PBS for 2 h at RT. After incubation, cells were washed thrice with PBS and incubated with Alexa Fluor 488 goat anti-mouse (1:500, Life Technologies; # A11001) in 3% BSA in PBS for 1 h at RT. After three times washing with PBS, cells were stained for actin using Alexa Fluor® 633 phalloidin dye (3 units; Life Technologies; #A22284) for 10 min at RT, then cells were washed again with PBS and further stained with 0.2-μM Hoechst (Thermo Fisher Scientific, #H3570) for 10 min at RT. Subsequently, cells were again washed with PBS and coverslips with fixed cells were mounted on glass slides using ProLong® Diamond Antifade (Thermo Fisher Scientific, #P36934). Cells were examined under a confocal microscope (Leica TCS SP5 II; Leica Microsystems, Wetzlar, Germany), and brightness adjustment of all images was done by using Leica Application Suite Advanced Fluorescence version 2.7.3.9723 software.

### Statistical analysis

All experiments were done at least thrice, and results were denoted as mean ± standard error of the mean (SEM). Statistical analysis was performed using one-way ANOVA for all the experiments, and *p* ≤ 0.05 was considered to be statistically significant.
