# Supplementary material for: BST2 regulates interferon gamma-dependent decrease in invasion of HTR-8/SVneo cells via STAT1 and AKT signaling pathways and expression of E-cadherin
Source: Cell Adh Migr. 2020 Jan 20;14(1):24–41. doi: 10.1080/19336918.2019.1710024 (PMC6973314; doi:10.1080/19336918.2019.1710024)
Supplement: Supplemental Material [file kcam-14-01-1710024-s001.docx]

**BST2 regulates interferon gamma-dependent decrease in invasion of HTR-8/SVneo cells via STAT1 and AKT signaling pathways and expression of E-cadherin**

Sonam Verma^1^, Amandeep Kaur Kang^1#^, Rahul Pal^2^, Satish Kumar Gupta^1^*

**Address for Correspondence**

Dr. Satish Kumar Gupta, PhD

Emeritus Scientist

J. C. Bose National Fellow

Reproductive Cell Biology Laboratory

National Institute of Immunology

Aruna Asaf Ali Marg

New Delhi-110 067

India

Tel: +91 11 26741249

Fax: +91 11 26742125

Email: [skgupta@nii.ac.in](mailto:skgupta@nii.ac.in)


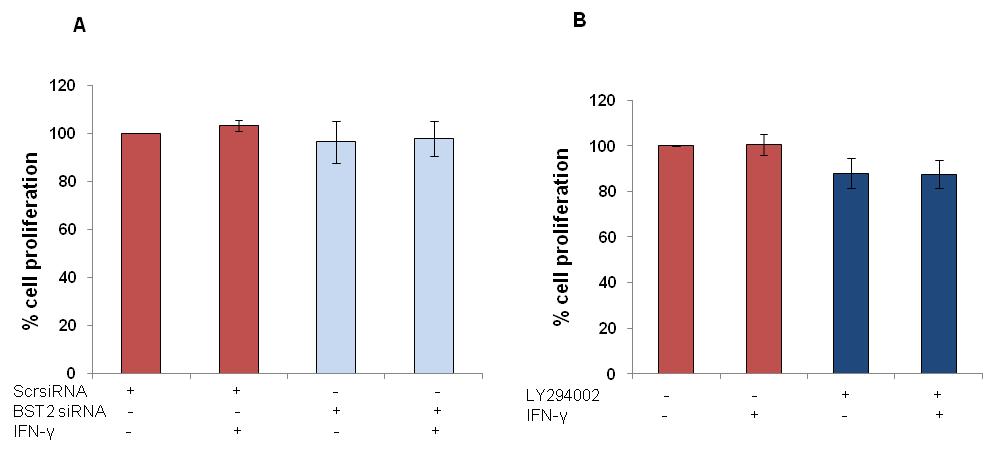


**Suppl Fig.** **1 - Effect of BST2 silencing by siRNA and inhibition of AKT by LY294002 pre-treatment on the proliferation of HTR-8/SVneo cells in the presence of IFN-γ.** HTR-8/SVneo cells (0.1 X 10^6^/well) were seeded in 6-well cell culture plate and incubated overnight at 37°C in 5% CO_2_ and 70% relative humidity. Next day, cells were transfected with either scrambled siRNA or BST2 siRNA for 48 h. Subsequently, cells were trypsinized, counted and 8000 cells/well were seeded in 96-well cell culture plates in the presence or absence of IFN-γ for 24 h at 37ºC in 5% CO_2_ & 70% relative humidity and used to study their proliferation by BrdU incorporation. In another experiment, HTR-8/SVneo cells pre-treated with or without LY294002 and subsequently treated with IFN-γ for 24 h as described above, were also processed for BrdU incorporation. **Panel A** shows the percent cell proliferation transfected with either scrambled siRNA or BST2 siRNA and subsequently treated with and without IFN-γ. **Panel B** shows the percent cell proliferation in control and LY294002 pre-treated cells in the presence and absence of IFN-γ treatment. Percent cell proliferation was calculated from normalized absorbance (absorbance in experimental group - absorbance of blank wells) values of untreated group divided by treated group and multiplied by 100.


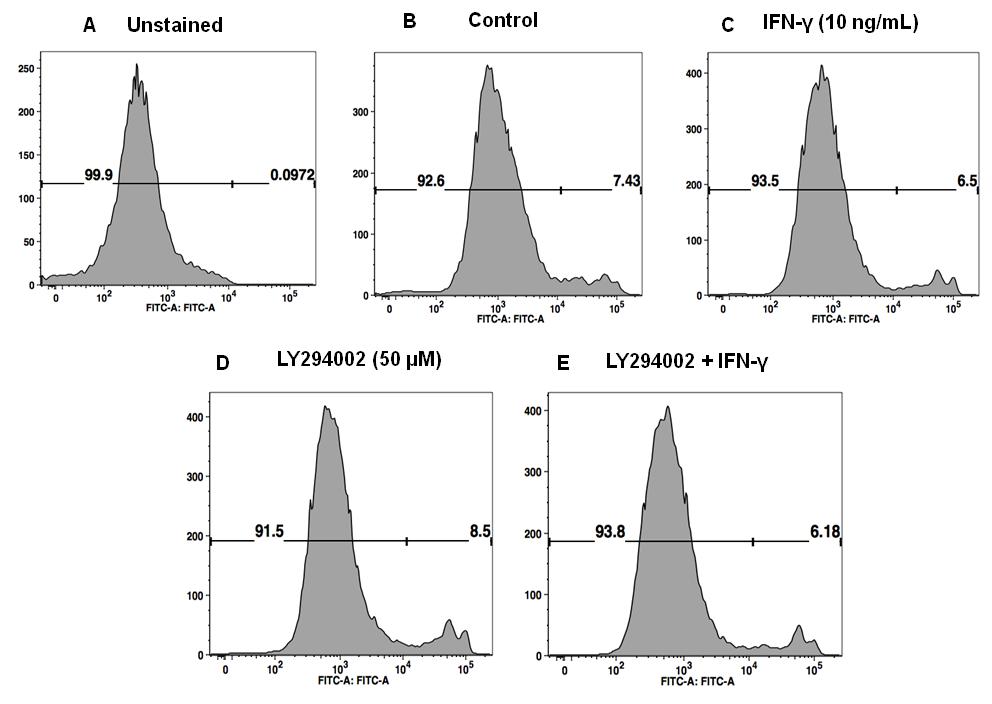


**Suppl Fig. 2 - Effect of pre-treatment with LY294002 (PI3K inhibitor) on the cell viability of HTR-8/SVneo cells subsequently treated with IFN-γ.** HTR-8/SVneo cells (0.1 x 10^6^/well) were cultured in 6-well cell culture plate overnight at 37°C in 5% CO_2_ and 70% relative humidity. Next day, cells were pre-treated with and without LY294002 (50 μM) for 2 h followed by treatment with and without IFN-γ (10 ng/mL) for 24 h and cell viability assay (SYTOX^TM^ green staining) was performed as described in *Materials and Methods.* The cells were trypsinized and re-suspended in saline (0.9 % Nacl) with working concentration of 5 μM SYTOX^TM^ green. **Panels A, B, C, D** & **E** show the profiles of live and dead cells in unstained, untreated control, IFN-γ treated, LY294002 (PI3K inhibitor) pre-treated, and LY294002 (PI3K inhibitor) pre-treated followed by IFN-γ treatment in HTR-8/SVneo cells respectively. Percent total unstained (live cells) and stained cells (dead cells) were calculated by Flow Cytometry Facs Verse.


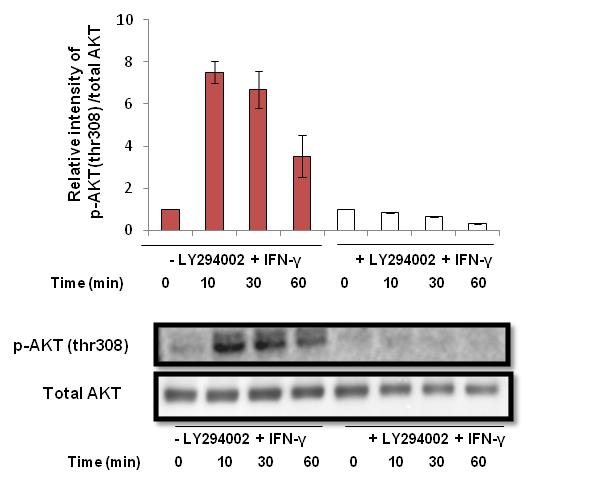


**Suppl Fig. 3 - Confirmation of AKT inhibition by LY294002 in HTR-8/SVneo cells treated with IFN-γ.** HTR-8/SVneo cells (0.1 x 10^6^/well) were cultured in 6-well cell culture plate overnight at 37°C in 5% CO_2_ and 70% relative humidity. Next day, cells were pre-treated with and without LY294002 (50 μM) for 2 h followed by treatment with IFN-γ (10 ng/mL) for 0, 10, 30 and 60 min. After treatment, cell lysates were prepared and proteins resolved by 0.1% SDS-10% PAGE and processed for analysis of phosphorylated form of AKT (thr308) and total AKT by Western blotting as described in *Materials and Methods.* The densitometric plot shows relative intensity of p-AKT (thr308) with respect to total AKT in untreated and LY294002 pre-treated cells, which were subsequently treated with IFN-γ. Representative blots of p-AKT (thr308) and total AKT are appended. The data is expressed as fold change with respect to 0 min control of respective treatment set and values are shown as mean ± S.E.M. of at least three independent experiments.


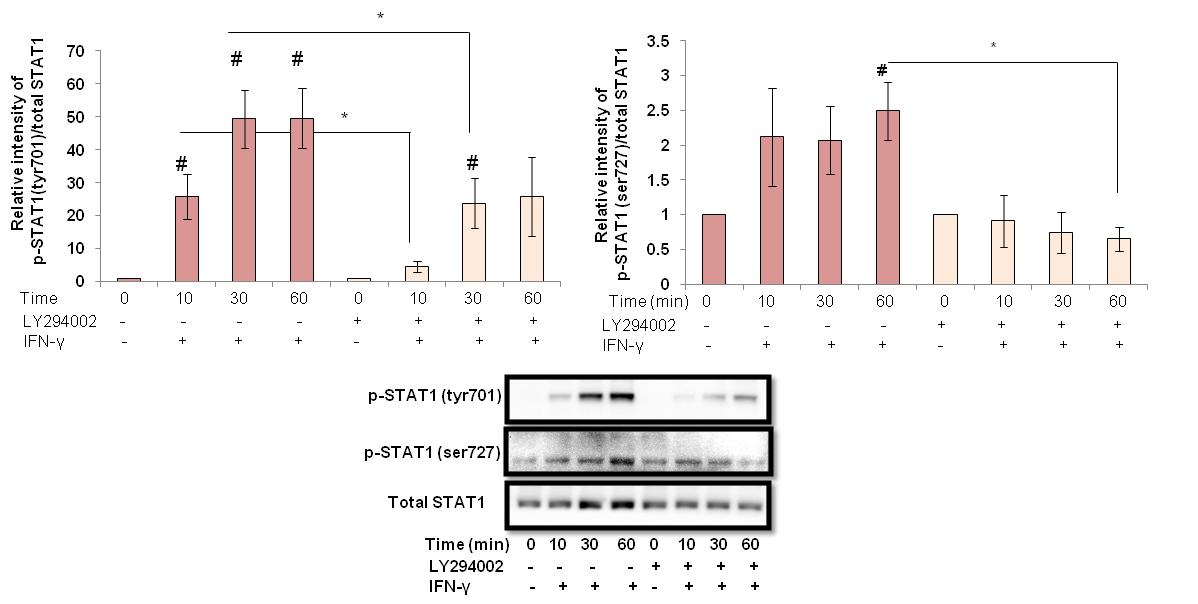


**Suppl Fig. 4 - Effect of LY294002 pre-treatment on the activation of STAT1 in the presence of IFN-γ**. HTR-8/SVneo cells (0.1 x 10^6^/well) were cultured in 6-well cell culture plate overnight at 37°C in 5% CO_2_ and 70% relative humidity. Next day, cells were pre-treated with LY294002 (50 μM) for 2 h followed by treatment of IFN-γ (10 ng/mL) for 10, 30 and 60 min as described in *Materials and Methods*. **Panels A and B** show the densitometric profile of p-STAT1 (tyr701) and p-STAT1 (ser727) in cells pre-treated with and without LY294002 in the presence of IFN-γ normalized using total STAT1. Representative blots of p-STAT1 (tyr701), p-STAT1 (ser727) and total STAT1 are appended as **Panel C**. ^#^ Represents the significant p value (≤ 0.05) in IFN-γ treated cells for 10, 30, 60 min as compared to 0 min with and without pre-treatment of LY294002. *Represents the significant p value (≤ 0.05) in LY294002 pre-treated group *vs* LY294002 untreated group in the presence of IFN-γ.
